# Oxygen Gas and UV Barrier Properties of Nano-ZnO-Coated PET and PHBHHx Materials Fabricated by Ultrasonic Spray-Coating Technique

**DOI:** 10.3390/nano11020449

**Published:** 2021-02-10

**Authors:** Mohsin Abbas, Mieke Buntinx, Wim Deferme, Naveen Reddy, Roos Peeters

**Affiliations:** 1 IMO-IMOMEC, Packaging Technology Center, Hasselt University, Wetenschapspark 27, 3590 Diepenbeek, Belgium; mieke.buntinx@uhasselt.be (M.B.); naveen.reddy@uhasselt.be (N.R.); roos.peeters@uhasselt.be (R.P.); 2 IMO-IMOMEC, Functional Materials Engineering, Hasselt University, Wetenschapspark 1, 3590 Diepenbeek, Belgium; wim.deferme@uhasselt.be; 3 IMEC vzw, Division IMOMEC, Wetenschapspark 1, 3590 Diepenbeek, Belgium

**Keywords:** zinc oxide nanoparticles, ultrasonic spray-coating, polyethylene terephthalate, poly(3-hydroxybutyrate-*co*-3-hydroxyhexanoate), oxygen transmission rate, UV barrier properties

## Abstract

Ultrasonic spray-coating (USSC)—a wet chemical deposition method to deposit ultrathin (down to 20 nm) coatings—is being applied as a promising alternative deposition method for functional coatings due to an economical, simple, and precise coating process with easy control over its operating parameters. In this research, zinc oxide nanoparticles (ZnO NPs) were ultrasonically spray-coated on commercial-grade polyethylene terephthalate (PET) and poly(3-hydroxybutyrate-*co*-3-hydroxyhexanoate) (PHBHHx) films. The most suitable parameters for the ink composition, the ultrasonic spray-coating process, and the number of coating passes (up to 50×) were selected on the basis of a series of experiments. The oxygen gas barrier properties in terms of the oxygen transmission rate (OTR) of neat PET, and 3×, 5×, 10×, and 50× ZnO NP-coated PET and PHBHHx substrates were investigated. The OTR values for neat PET, and 3×, 5×, and 10× ZnO NP-coated PET substrates were found to be the same; however, a 5% reduction in OTR for 50× ZnO NP-coated PET substrate was observed compared to the neat PET substrate. No reduction in OTR was found for any above number of coating passes on PHBHHx substrates against the neat PHBHHx substrate. However, the ultraviolet (UV) tests of 3×, 5×, and 10× ZnO NP-coated PET and PHBHH× substrates revealed a significant decrease in percentage transmission for 10× coated PET and PHBHHx substrates as compared to their 3× and 5× ZnO NP-coated substrates, respectively. It was revealed from the study that the 50× ZnO NP coating of the PET substrate created a slight difference in OTR as compared to the reference substrate. However, the ultrasonic spray-coating method created a significant UV barrier effect for 3×, 5×, and 10× ZnO NP-coated PET and PHBHHx substrates, which demonstrates that the optimized coating method cannot be used to create a high oxygen barrier but can certainly be applied for UV barrier applications in food packaging. It is concluded that ultrasonic spray deposition of ZnO NPs on PET and PHBHHx materials has shown promising results for UV barrier properties, demonstrating the advantages of using this method compared to other coating methods with regard to cost-effectiveness, precise coating, and better process control.

## 1. Introduction

In the field of food packaging, nanomaterials are creating an incredible impact by improving the multifunctional properties of packaging films [1]. Many reports focused on applications of nanomaterials concerning food quality assurance and improvements in food packaging [2,3,4,5,6]. For example, silver nanoparticles (Ag NPs) can protect food from microbial invasion [7]. Titanium dioxide (TiO_2_) nanoparticles can be used as an ultraviolet (UV) barrier in food packaging. These nanoparticles have also been utilized due to their antimicrobial activity [8]. Copper (Cu) and copper oxide (CuO), cadmium (Cd), zinc oxide (ZnO), magnesium oxide (MgO), and single-walled carbon nanotubes (SWCNTs) are also reported for their antimicrobial activity [9]. Amongst these nanoparticles, ZnO NPs have achieved a significant position in enhancing the packaging properties such as mechanical, barrier, and antimicrobial properties [10]. Using these nanoparticles in packaging polymers is generally possible through two routes: by mixing or dispersing the nano-objects within a polymer matrix or by applying nanocoatings on the surface of the polymers [11]. In the first approach, packaging films consisting of ZnO NPs along with (bio)polymers have been made through diverse processing techniques, such as melt compounding [12], solvent casting [13,14], twin-screw extrusion [15], solution casting [16,17,18,19], and extrusion blow molding [20], leading to the evaluation of their gas barrier and mechanical properties. As far as the second approach is concerned, coatings in the packaging sector have shown incredible growth in recent years. This has become possible due to two main factors: (1) increased accessibility of various types of nanoparticles, and (2) progress and innovations in the processes capable of controlling the coating structure. Various types of coatings are available in food packaging applications, such as nanocoatings inside the packaging, outside the packaging, in the form of a layer sandwiched in multilayer packaging, coatings on polymers with high barrier properties, and application of edible coatings on a variety of foods serving as lipid, moisture, and gas barriers [21]. The coating processes applied are pulsed laser deposition, vapor deposition, magnetron sputtering, plasma-assisted/ion-beam-assisted techniques, layer-by-layer coating, sol–gel coating, dip coating, electrochemical deposition, electrospinning, and electrospraying techniques [22,23]. Nanocoatings have proven to be incredibly pertinent in surface functionalization to provide essential properties such as gas barrier [24], antimicrobial [25], flame retardant [26], and self-healing [27] properties.

A 50 nm coating of ZnO NPs deposited on the surface of polyethylene naphthalate (PEN) by radio frequency (RF) magnetron sputtering decreased the oxygen permeability to <5 (mL/m^2^·day·atm)—almost eightfold lower oxygen permeability as compared neat PEN [28]. ZnO NP coatings have also been used to protect PEN against ultraviolet (UV) light [28,29]. Furthermore, ZnO NPs have also been used for their photocatalytic effect [30,31,32]. ZnO NP-loaded starch-coated polyethylene films have been produced to improve antibacterial properties [33].

In this study, we used two different kinds of materials, a synthetic polymer, polyethylene terephthalate (PET), and a biopolymer, poly(3-hydroxybutyrate-*co*-3-hydroxyhexanoate) (PHBHHx). PET is a commercially available thermoplastic [34] and a highly demanding packaging material originating from a petrol-based source. Due to nondegradable behavior, these synthetic polymers cause environmental pollution and adversely affect the wildlife system. Due to these drawbacks, ecofriendly, nontoxic, and biodegradable polymers are attaining more attention from researchers. These biopolymers are more sustainable alternatives to synthetic polymers. However, their barrier and mechanical properties are not so good as those of synthetic polymers [35]. PHBHHx, a biopolymer, is a member of the polyhydroxyalkanoate (PHA) family [36]. It is an emerging biopolymer that is an environmentally friendly material with food packaging applications [37,38].

**Figure 1:**
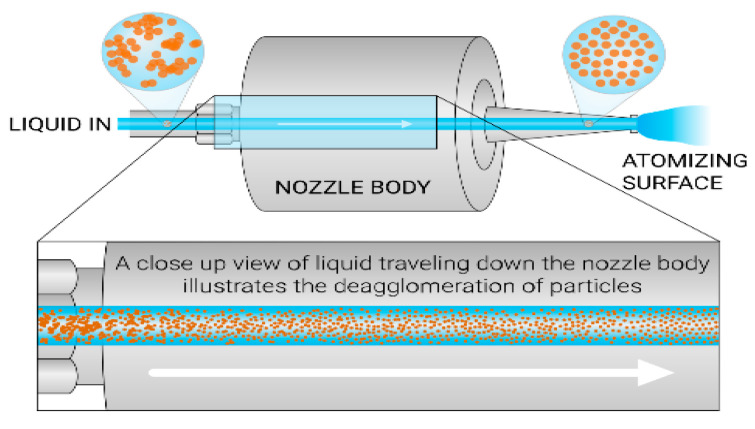
An illustration of an atomization process in the ultrasonic spray nozzle [43].

There are various conventional coating technologies to coat nanoparticles on substrates. Their uniformity control is limited, and nanomaterial consumption is also excessive and costly [39]. In this study, an ultrasonic spray-coating method was chosen to deposit ZnO NPs on PET and PHBHHx materials due to the following reasons: process simplicity, precise coating, economical, good transfer efficiency, good reproducibility, and production of droplets in the micrometer range [40,41]. Ultrasonic spray-coating (USSC) works on the principle of ultrasonic atomization. In this technique, high-frequency sound (ultrasonic) vibrations generate a fine mist of the coating solution [42], which leads to a narrow distribution in size and composition of the generated droplets, as illustrated in Figure 1. In contrast, a broad distribution is usually obtained in the conventional spray-coating.

The coating material and process parameters must be controlled to obtain a uniform layer with ultrasonic spray-coating. The critical factors that need to be emphasized are as follows: the ink concentration, the solvents used, the substrate used for spraying, the flow rate of the solution, the speed of the nozzle while spraying, the height of the nozzle from the substrate, the number of passes, the temperature of the hot plate on which substrate is placed, and the inert gas guiding pressure which leads the droplets toward the substrate.

This research focused on depositing ZnO nanoparticles on commercial-grade PET and self-made PHBHHx substrates by optimizing the ultrasonic spray-coating method to obtain coated materials with optimized oxygen gas and UV barrier properties.

## 2. Materials and Methods

### 2.1. Materials

#### 2.1.1. Ink Materials

ZnO nanoparticles (20 wt.% dispersion in water, <100 nm particle size (transmission electron microscope—TEM), ≤40 nm average particle size (APS)), and polyvinyl alcohol (PVA) with average molecular weight (M_W_) = 146,000–186,000, 99+% hydrolyzed, crystalline powder were purchased from Sigma Aldrich, USA. Laboratory-grade isopropyl alcohol (IPA) and deionized (DI) water were also used in preparing the solution.

#### 2.1.2. Substrates

##### Commercial-Grade PET Foils

These foils were purchased from DuPont Teijin films with brand Melinex ST506, 125 µm thick polyester film. The PET sheet was cut into dimensions of 3.5 cm × 3.5 cm for making samples. These samples were cleaned to remove dirt and contaminations. The beaker was washed with DI water and then dried with N_2_ air. Then, PET samples were immersed in the beaker containing a soapy water solution, and this beaker was placed in an ultrasonication bath for 15 min. Then, the PET samples were put in the beaker containing DI water and were sonicated for 15 min. This step was again repeated with fresh DI water. PET samples were soaked with acetone for few seconds and then were immersed in the beaker containing IPA. This beaker was placed in an ultrasonication bath for 15 min of sonication. Then, the PET samples were taken out of the beaker and dried with N_2_ air.

##### Compression-Molded PHBHHx Films

The PHBHHx granulates were kindly provided by Kaneka Corporation, Belgium. The PHBHHx samples with 0.5 mm thickness were prepared from their granulates using a Collin Plate Press 200 E machine, which was kindly provided by Catholic University Leuven (KU Leuven) in Belgium. These granulates were placed on a stainless-steel mold, and then one Teflon sheet and one stainless-steel plate were placed above the mold, while a second set was placed below the mold. This sample set was placed between the plates of the Plate Press. The temperature of both plates was kept at 160 °C for 10 min without any pressure on the plates. The pressure was raised to 30 bar for 2 min. This pressure was slowly increased to 150 bar for 2 min. The pressure was reduced to zero, and the temperature of both plates was lowered to 60 °C. Then, the pressure was increased to 50 bar for 20 min. Then, heating was stopped, and the mold with PHBHHx sample was removed from the Plate Press, which led to the ultrasonic spray-coating step.

### 2.2. Methods

#### 2.2.1. Ultrasonic Spray-Coating

An ExactaCoat ultrasonic spray-coating system from Sono-Tek (Milton, NY, USA) equipped with an AccuMist™ ultrasonic spraying nozzle was used for the coating experiments in this article. In the ultrasonic spray-coating process, the main parameters that need to be considered are the following:

- Ink composition: A solution prepared with nanoparticles along with chemicals, including solvents.
- Ink flow rate (mL/min): The volume of the ink sprayed per unit time [44].
- Path speed (mm/s): The distance traveled by the ultrasonic spray nozzle per unit time.
- Nozzle frequency (kHz): This is the frequency at which the nozzle vibrates [39].
- Generator power (W): This is the power required by the generator to operate the ultrasonic spray nozzle [45].
- Hot-plate temperature (°C): The value of the temperature set for the hot plate on which the substrate is placed.
- Nozzle to substrate distance (mm): This is the distance between the nozzle tip and the substrate.
- Nitrogen guiding pressure (kPa): This gas pressure helps move the sprayed droplets toward the substrate [46].
- The number of coated layers: This is the number of layers coated on the substrates.

#### 2.2.2. Surface Morphology

An optical microscope, Nikon Eclipse ME600, was used for the optical characterization of the 3×, 5×, and 10× ZnO NP-coated PET and PHBHHx substrates, respectively. A 20× resolution of the instrument was set for optical microscopic characterization of these substrates. The surface morphology of 50× ZnO NP-coated PET and PHBHHx substrates was examined using an FEI Quanta 200F scanning electron microscope (SEM), USA. Backscattered electrons (BSE) detection mode was used in the image formed for surface morphological characterization. Backscattered electrons are those electrons that scatter backward and emit out of the sample. In the BSE detection mode, information from the deep region can be obtained [47]. Top-side and cross-sectional images of these coated substrates were analyzed.

Moreover, an atomic force microscope (AFM, NX10—Park Systems, Suwon, South Korea) was also used to obtain the topographic structural characterization of the ZnO NP-coated substrate. The AFM characterization was performed using an ACTA probe in the tapping mode of the AFM instrument. The scan area of the sample was kept as 25 µm × 25 µm.

#### 2.2.3. Gas Barrier Characterization

A Mocon Ox-Tran^®^ was used to measure the oxygen transmission rates (OTR, cc/m^2^·day) of neat and 3×, 5×, 10×, and 50× ZnO NP-coated PET and PHBHHx substrates. The OTR tests for each sample were performed in triplicate. Thirty samples in total were prepared in aluminum masks with a surface area of 5 cm^2^. The operating conditions of OTR tests were maintained at 23 °C and 0% relative humidity (RH). In these tests, the samples were exposed to oxygen on one side and carrier gas (nitrogen/helium) on the other side, keeping both sides at a pressure of 1 atm. Thickness measurements were done to calculate the oxygen permeability coefficients of neat PET and PHBHHx samples using an MTS MI20 thickness gauge instrument. The thickness value of each sample was taken as an average of five measurements at different locations.

#### 2.2.4. UV Barrier Characterization

A Carry 5000 UV/visible light (Vis)/near-infrared (NIR) spectrophotometer (Agilent Technologies, Santa Clara, CA, USA) in scan mode with a resolution of 1 nm was used for UV characterization of the eight samples—neat PET and 3×, 5×, and 10× ZnO NP-coated PET and PHBHHx samples. Initially, the PET and PHBHHx materials were cut into dimensions of 2.5 cm × 2.5 cm. The UV tests were performed in terms of percentage transmission with a wavelength (λ) ranging from 200 nm to 800 nm.

## 3. Results and Discussion

### 3.1. Coating Parameters

First of all, a series of experiments were performed to find the ink composition of 2.5 wt.% in PVA, IPA, and DI water, which was suitable to obtain a uniform ink flow pattern of the ultrasonic spray coater. Several experiments were conducted on the basis of carefully selected parameter combinations. The combination that gave the best results regarding uniform coating is presented in Table 1.

The PET and PHBHHx substrates were spray-coated with 3×, 5×, 10×, and 50× coated layers of ZnO NPs, respectively. The whole ultrasonic spray-coating process was conducted through a controlled and three-dimensional (3D) programable system. All samples were prepared in triplicate for OTR analysis.

### 3.2. Optical Microscopic (OM) and Scanning Electron Microscopic (SEM) Characterizations

**Figure 2:**
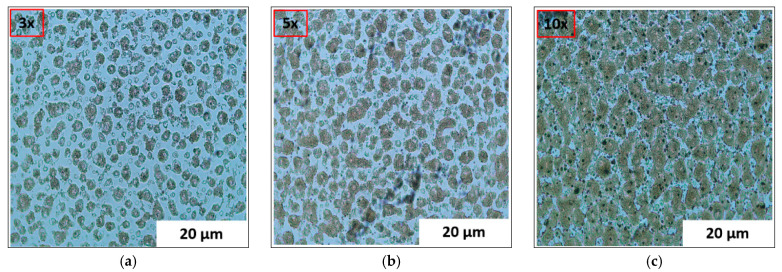
(**a**) Optical microscopic (OM) image of 3× ZnO nanoparticle (NP)-coated polyethylene terephthalate (PET) substrate; (**b**) OM image of 5× ZnO NP-coated PET substrate; (**c**) OM image of 10× ZnO NP-coated PET substrate.

**Figure 3:**
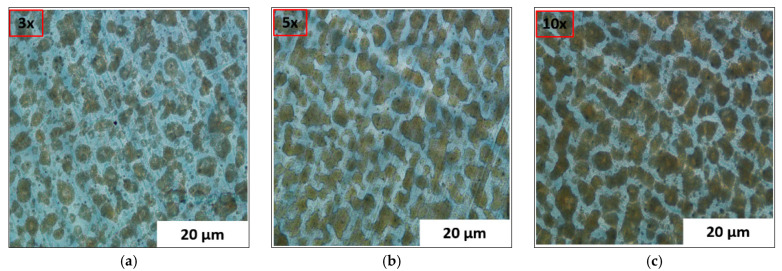
(**a**) OM image of 3× ZnO NP-coated poly(3-hydroxybutyrate-*co*-3-hydroxyhexanoate) (PHBHHx) substrate; (**b**) OM image of 5× ZnO NP-coated PHBHHx substrate; (**c**) OM image of 10× ZnO NP-coated PHBHHx substrate.

The optical microscopic (OM) characterization of 3×, 5×, and 10× ZnO NP-coated PET and 3×, 5×, and 10× ZnO NP-coated PHBHHx substrates revealed that the surface coverage of ZnO NPs on these substrates was increased upon increasing the number of coated layers, as shown in Figure 2a–c and Figure 3a–c, respectively.

**Figure 4:**
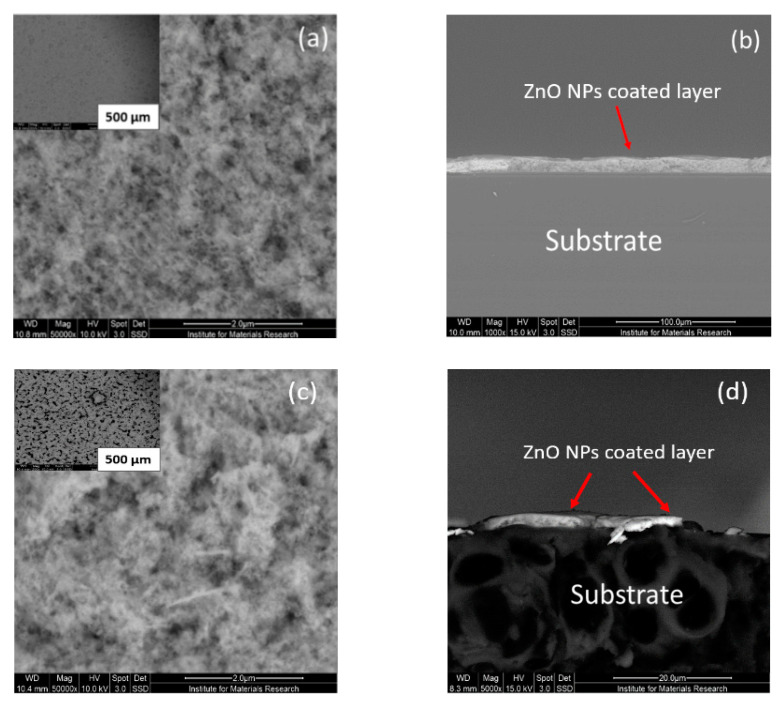
(**a**) Top-side SEM image of 50× ZnO NP-coated PET substrate; (**b**) cross-sectional SEM image of 50× ZnO NP-coated PET substrate; (**c**) top-side SEM image of 50× ZnO NP-coated PHBHHx substrate; (**d**) cross-sectional SEM image of 50× ZnO NP-coated PHBHHx substrate [48].

As concluded from the OM images of ZnO NP-coated PET and PHBHHx substrates, further experiments were conducted to increase the number of coated layers to enhance the surface coverage of these substrates. Therefore, after ultrasonic spray-coating of ZnO NPs on both substrates by 50× coated layers, these samples were characterized by scanning electron microscopy to investigate the structure of the top-coated layers. Initially, the morphology of a 50× ZnO NP-coated PET substrate from its top and cross-sectional views was analyzed by SEM. An evenly and nicely coated layer was observed in the 50× ZnO NP-coated PET substrate as depicted from Figure 4a,b, respectively. In Figure 4b, the ZnO NP-coated layer is indicated by a red arrow, and the PET substrate is shown below the coated layer. On the other hand, a non-homogenously coated surface was found in the case of PHBHHx substrates, as shown in Figure 4c,d, respectively. These images showed that ZnO NPs poorly adhered to the original PHBHHx substrate, as also depicted in the cross-sectional image shown in Figure 4d. In Figure 4d, the ZnO NP-coated layer is highlighted by a red arrow under which the PHBHHx substrate is shown. It can also be seen that ZnO NPs were not well adhered to the surface of the PHBHHx substrate, leaving its surface noncoated due to the hydrophobic behavior of the PHBHHx material itself [35].

### 3.3. AFM Characterization

**Figure 5:**
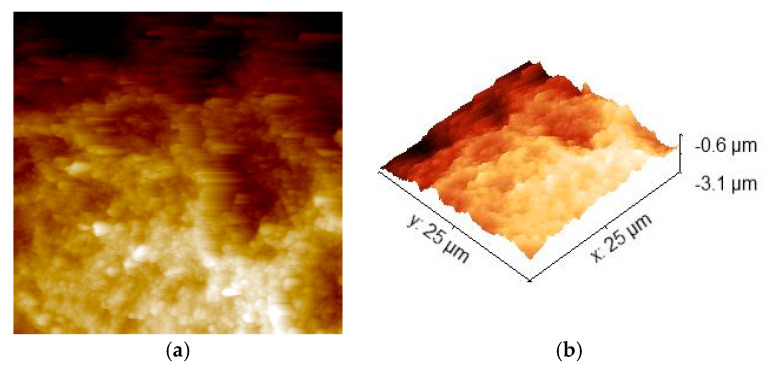
(**a**) Topographic atomic force microscopy (AFM) image of 25 µm × 25 µm scan area of 50× ZnO NP-coated PET substrate; (**b**) three-dimensional (3D) AFM image of 25 µm × 25 µm scan area of 50× ZnO NP-coated PET substrate.

As revealed from the Figure 4c,d, the ZnO NPs were not attached to the surface of the 50× coated PHBHHx substrate; therefore, AFM characterization of 50× ZnO NP-coated PET substrate was performed. Figure 5a represents the topography of the 25 µm × 25 µm scanned area of the sample, while Figure 5b represents the 3D image of this area. In the AFM characterization, it was observed from the topographic image that the surface of the PET substrate was covered with ZnO NPs, as shown in Figure 5a; however, a variation was observed in the surface profile of the coated layer, as shown in Figure 5b. The white parts of the coated layer indicated the less dense coating of ZnO NPs on the PET substrate. The dark portions of these images represent the presence of more densely coated ZnO NPs. These results were in good agreement with the SEM results, as shown in Figure 4a,b.

### 3.4. OTR

**Scheme 1:**
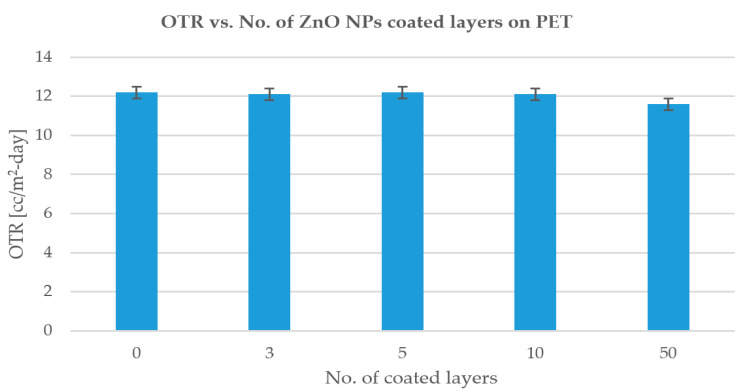
The comparison between oxygen transmission rate (OTR, cc/m^2^·day) and number of ZnO NP-coated layers on PET substrates.

PET substrates: The OTR values of neat PET, 3×, 5×, and 10× ZnO NP-coated PET substrates were 12.2 ± 0.3, 12.1 ± 0.4, 12.2 ± 0.2, and 12.1 ± 0.3 cc/m^2^·day, respectively, while the OTR values of 50× ZnO NP-coated PET substrates were observed as 11.6 ± 0.2 cc/m^2^·day, as shown in Scheme 1. The given mean values were calculated for three samples each for neat and 3×, 5×, 10×, and 50× coated PET substrates. The thickness value of the neat PET sample was 0.13 ± 0.002 mm, while the thickness of the coated layer on the 50× ZnO NP-coated PET substrate was found to be 10.4 µm. The permeability coefficient (PO_2_) (cc·mm/m^2^·day) is the product of OTR (cc/m^2^·day) and thickness (mm) of the sample [1]. The PO_2_ value of the neat PET substrate was obtained as ~1.6 cc·mm/m^2^·day, as also mentioned in the literature [37]. Due to the very minute value of coating thickness, permeability coefficients of neat and 50× ZnO NP-coated PET substrates did not have a considerable difference, which also applied to the remaining substrates. As shown in Figure 4a,b, the 50× ZnO NP-coated PET substrate exhibited some hindrance to the flow path of small oxygen molecules by creating a tortuous path [49] on top of the material. This tortuous path is typically created by a well homogeneously ZnO NP-coated layer on the substrate to restrict the movement of oxygen gas molecules. In this study, due to the insignificant tortuous path, an overall ~5% decrease in the OTR of the 50× ZnO NP-coated PET substrate compared to the neat PET was observed. However, micro-cracks and open noncoated spots could be the reason for not creating a considerable difference between the OTR values of neat PET and 50× ZnO NP-coated PET substrate. On the other hand, increasing the number of coated layers can also cause the creation of agglomerates on the surface of substrates and the simultaneous settling down of nanoparticles in the ink syringe, which can clog the nozzle tubing. Therefore, pretreatment of the reference substrate could lead to an improvement of the oxygen gas barrier properties.

**Scheme 2:**
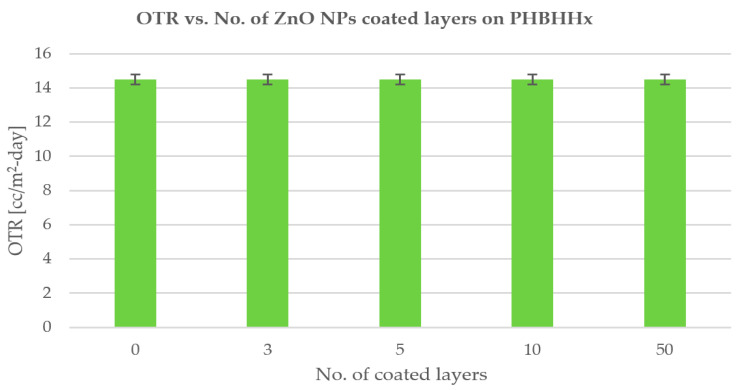
The comparison between OTR (cc/m^2^·day) and number of ZnO NP-coated layers on PHBHHx substrates

PHBHHx substrates: The OTR values of neat PHBHHx and 3×, 5×, 10×, and 50× ZnO NP-coated PHBHHx substrates were found to be 14.5 ± 0.3, 14.4 ± 0.2, 14.5 ± 0.4, 14.6 ± 0.2, and 14.5 ± 0.3 cc/m^2^·day, respectively, as shown in Scheme 2. The given mean values were calculated for three samples each for neat and 3×, 5×, 10×, and 50× coated PHBHHx substrates. The thickness of the neat PHBHHx sample was 0.52 ± 0.1 mm, while the thickness of the 50× ZnO NPs coated layer was obtained as 1.4 µm. The permeability coefficient of neat PHBHHx material was obtained as ~8 cc·mm/m^2^/day, which is in close accordance with the literature [37]. Due to the negligible value of the coating thickness, permeability coefficients of neat and 50× ZnO NP-coated PHBHHx substrates did not have a substantial difference, which also applied to the remaining PHBHHx substrates. In the case of the 50× ZnO NP-coated PHBHHx substrate, the ZnO NP-coated layer could not adhere well to its top surface, as shown in Figure 4c,d. This behavior is also presented in the form of a graph plotting OTR vs. the number of ZnO NP-coated layers on PHBHHx films, where no difference in OTR can be seen. This non-adherence could be due to the hydrophobic behavior of the PHBHHx material itself, as mentioned in the literature [35,50]; consequently, the removal of this coated layer from the substrate caused no decrease in the OTR value.

### 3.5. UV/Vis Transmission Measurements

**Scheme 3:**
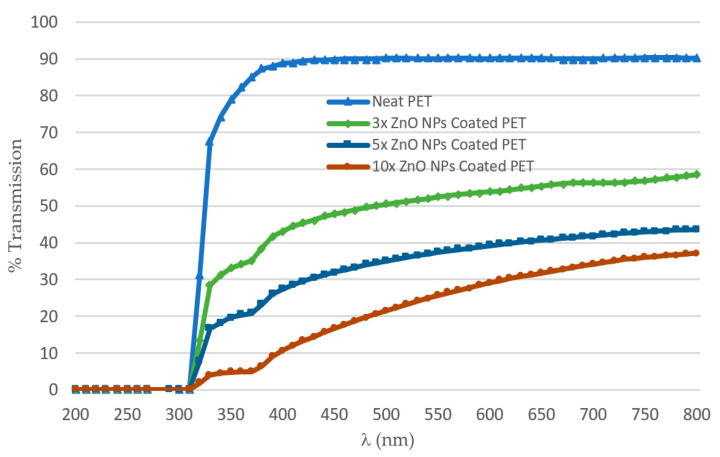
Ultraviolet (UV)/visible light (Vis) spectrum (percentage transmission vs. wavelength (nm)) of neat PET and 3×, 5×, and 10× ZnO NP-coated PET substrates.

Transmittance (T) is the fraction of incident light transmitted through a sample [51]. The purpose of the UV/Vis spectroscopic study in transmission mode was to determine how ZnO NPs could effectively create a UV blockage for neat PET, neat PHBHHx, ZnO NP-coated PET, and ZnO NP-coated PHBHHx substrates. One UV measurement per substrate was obtained in terms of percentage transmission and wavenumber (nm). As shown in Scheme 3, the ZnO NP absorption peak for 3×, 5×, and 10× ZnO NP-coated PET substrates was at 370 nm in the UVA region, which is in accordance with the literature, also indicating an almost sharp absorbance peak for ZnO NPs at 370 nm [52]. The energy band gap was found as ~3.3 eV. The transmission spectrum obtained for neat PET was also similar to that mentioned in the literature [53].

The UV transmission trend was in the following order: neat PET > 3× > 5× > 10×. This sequence demonstrates that a neat PET substrate transmitted maximum UV light, while the lowest transmission was possible for a 10× coated layer PET substrate, which shows that increasing the number of coated layers could reduce the UV transmission and increase the UV absorbance. Scheme 3 shows that the percentage transmission for the 10× ZnO NP-coated PET substrate was below 40% at the wavelength of 800 nm. Therefore, it was expected from the UV experiments performed on 3×, 5×, and 10× ZnO NP-coated PET substrates that the percentage transmission of the 50× ZnO NP-coated PET substrate would be almost zero. Hence, the percentage transmission of the 50× ZnO NP-coated PET substrate is not included.

**Scheme 4:**
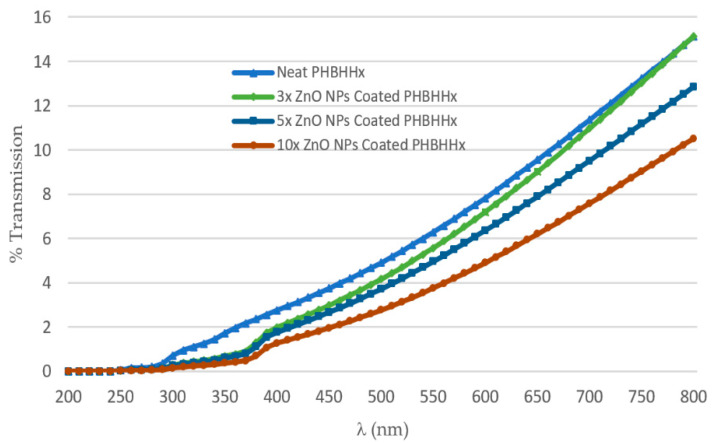
UV/Vis spectrum percentage transmission vs. wavelength (nm)) of neat PHBHHx and 3×, 5×, and 10× ZnO NP-coated PHBHHx substrates.

In Scheme 4, ZnO nanoparticles showed an absorption peak at 390 nm wavelength in the UVA region; however, this was not a sharp peak as seen for coated PET samples. The UV transmission trend was in the following sequence: neat PHBHHx > 3× > 5× > 10×. This order confirms that the higher number of coated layers would result in a more covered surface area and subsequently lower percentage transmission. The UV transmission results of the 50× ZnO NP-coated PHBHHx substrate are also not presented in the graph due to the expected near-zero percentage transmission. The reasons for the deficient UV transmission of PHBHHx samples are the following:

1. Self-made PHBHHx used was not a transparent material like PET material, i.e., commercial-grade foil.
2. The thickness of the self-made PHBHHx material was approximately 0.5 mm, while the PET material thickness was 0.13 mm. It was revealed that increasing the path length traveled by incident light caused less UV transmission, as also evident from Beer–Lambert’s law [54].

In both coated PET and coated PHBHHx substrates, the ZnO NPs created a significant shielding effect to UV radiation, and these ZnO NPs could extend the UV absorption area of the neat PET and PHBHHx films.

## 4. Conclusions

The ultrasonic spray-coating technique was adopted to deposit ZnO nanoparticles on PET and PHBHHx substrates. The most suitable ultrasonic spray-coating conditions were used, and 3×, 5×, 10×, and 50× layers were coated on the substrates. A uniformly covered layer of ZnO NPs for 50× ZnO NP-coated PET substrate was obtained via the ultrasonic spray-coating process, as evident from the scanning electron microscopy (SEM) images. The barrier properties of neat and coated samples were analyzed and compared. A slight difference in OTR (~5%) was observed between 50× ZnO NP-coated PET and neat PET or 3×, 5×, and 10× ZnO NP-coated PET substrates, while no difference in OTR was found for neat and all other ZnO NP-coated PHBHHx substrates. An improvement in gas barrier properties could be acquired through surface modifications such as the pretreatment of neat substrates with plasma and corona treatments. These treatments can significantly improve the gas barrier properties of coated materials. In future work, suitable pretreatment of neat substrates can be used to significantly enhance the barrier properties. Moreover, X-ray diffraction (XRD) and Fourier-transform infrared (FTIR) characterization measurements could help to further explore the surface morphology of the coated substrates.

Nonetheless, the UV/Vis spectroscopic results revealed that ZnO NPs created a good blocking layer against light in the UVA region. The 10× ZnO NP-coated PET substrate showed the highest UV barrier as compared to the 5× ZnO NP-coated PET, 3× ZnO NP-coated PET, and neat PET. A similar trend was also observed for PHBHHx substrates. Therefore, using the ultrasonic spray-coating technique with ZnO NPs could be a novel approach to produce UV barrier layers on other materials.

## Figures and Tables

**Table 1:** The optimized parameters of the ultrasonic spray-coater. PVA, polyvinyl alcohol; IPA isopropyl alcohol; DI, deionized.

Sr. No.	Parameters	Optimized Values
1	Ink composition	2.5 wt.% ZnO NP solution in PVA, IPA, and DI water
2	Path speed (mm/sec)	10 [44]
3	Nozzle frequency (kHz)	120
4	Ink flow rate (mL/min)	0.1
5	Generator power (W)	2.5 [44]
6	Nozzle to substrate distance (mm)	75
7	Hot plate temperature (°C)	30
8	Nitrogen guiding pressure (kPa)	0.34
9	Number of coated layers	3, 5, 10, and 50

## References

[1] Abbas M., Buntinx M., Deferme W., Peeters R. (2019). (Bio) polymer/ZnO nanocomposites for packaging applications: A review of gas barrier and mechanical properties. Nanomaterials.

[2] Wyser Y., Adams M., Avella M., Carlander D., Garcia L., Pieper G., Rennen M., Schuermans J., Weiss J. (2016). Outlook and Challenges of Nanotechnologies for Food Packaging. Packag. Technol. Sci..

[3] Ariyarathna I.R., Rajakaruna R., Karunaratne D.N. (2017). The rise of inorganic nanomaterial implementation in food applications. Food Control..

[4] Pathakoti K., Manubolu M., Hwang H.-M. (2017). Nanostructures: Current uses and future applications in food science. J. Food Drug Anal..

[5] Singh T., Shukla S., Kumar P., Wahla V., Bajpai V.K., Rather I.A. (2017). Application of Nanotechnology in Food Science: Perception and Overview. Front. Microbiol..

[6] Noruzi M. (2016). Electrospun nanofibres in agriculture and the food industry: A review. J. Sci. Food Agric..

[7] Cacciotti I., Fortunati E., Puglia D., Kenny J.M., Nanni F. (2014). Effect of silver nanoparticles and cellulose nanocrystals on electrospun poly(lactic) acid mats: Morphology, thermal properties and mechanical behavior. Carbohydr. Polym..

[8] Arora A., Padua G. (2010). Nanocomposites in food packaging. J. Food Sci..

[9] Arshak K., Adley C., Moore E., Cunniffe C., Campion M., Harris J. (2007). Characterisation of polymer nanocomposite sensors for quantification of bacterial cultures. Sens. Actuators B Chem..

[10] Huang Y., Mei L., Chen X., Wang Q. (2018). Recent Developments in Food Packaging Based on Nanomaterials. Nanomaterials.

[11] Iijima S. (1991). Helical microtubules of graphitic carbon. Nat. Cell Biol..

[12] Pantani R., Gorrasi G., Vigliotta G., Murariu M., Dubois P. (2013). PLA-ZnO nanocomposite films: Water vapor barrier properties and specific end-use characteristics. Eur. Polym. J..

[13] Arfat Y.A., Ahmed J., Al Hazza A., Jacob H., Joseph A. (2017). Comparative effects of untreated and 3-methacryloxypropyltrimethoxysilane treated ZnO nanoparticle reinforcement on properties of polylactide-based nanocomposite films. Int. J. Biol. Macromol..

[14] Jafarzadeh S., Ariffin F., Mahmud S., Alias A.K., Hosseini S.F., Ahmad M. (2017). Improving the physical and protective functions of semolina films by embedding a blend nanofillers (ZnO-nr and nano-kaolin). Food Packag. Shelf Life.

[15] Marra A., Silvestre C., Duraccio D., Cimmino S. (2016). Polylactic acid/zinc oxide biocomposite films for food packaging application. Int. J. Biol. Macromol..

[16] Díez-Pascual A.M., Díez-Vicente A.L. (2014). Poly(3-hydroxybutyrate)/ZnO Bionanocomposites with Improved Mechanical, Barrier and Antibacterial Properties. Int. J. Mol. Sci..

[17] Díez-Pascual A.M., Díez-Vicente A.L. (2014). ZnO-Reinforced Poly(3-hydroxybutyrate-co-3-hydroxyvalerate) Bionanocomposites with Antimicrobial Function for Food Packaging. ACS Appl. Mater. Interfaces.

[18] Venkatesan R., Rajeswari N. (2016). ZnO/PBAT nanocomposite films: Investigation on the mechanical and biological activity for food packaging. Polym. Adv. Technol..

[19] Ejaz M., Arfat Y.A., Mulla M., Ahmed J. (2018). Zinc oxide nanorods/clove essential oil incorporated Type B gelatin composite films and its applicability for shrimp packaging. Food Packag. Shelf Life.

[20] Ahmed J., Arfat Y.A., Al-Attar H., Auras R., Ejaz M. (2017). Rheological, structural, ultraviolet protection and oxygen barrier properties of linear low- density polyethylene films reinforced with zinc oxide (ZnO) nanoparticles. Food Packag. Shelf Life.

[21] Cagri A., Ustunol Z., Ryser E.T. (2004). Antimicrobial Edible Films and Coatings. J. Food Prot..

[22] Joshi M., Khanna R., Shekhar R., Jha K. (2011). Chitosan nanocoating on cotton textile substrate using layer-by-layer self-assembly technique. J. Appl. Polym. Sci..

[23] Caruso R.A., Antonietti M. (2001). Sol−Gel Nanocoating: An Approach to the Preparation of Structured Materials. Chem. Mater..

[24] Holder K.M., Spears B., Huff M.E., Priolo M.A., Harth E., Grunlan J.C. (2014). Stretchable Gas Barrier Achieved with Partially Hydrogen-Bonded Multilayer Nanocoating. Macromol. Rapid Commun..

[25] Xiao W., Xu J., Liu X., Hu Q., Huang J. (2013). Antibacterial hybrid materials fabricated by nanocoating of microfibril bundles of cellulose substance with titania/chitosan/silver-nanoparticle composite films. J. Mater. Chem. B.

[26] Li Y.-C., Mannen S., Cain A.C., Grunlan J.C. (2015). Flame retardant polymer/clay layer-by-layer assemblies on cotton fabric. Proc. Abstr. Papers Am. Chnem. Soc..

[27] Si Y., Zhu H., Chen L., Jiang T., Guo Z. (2015). A multifunctional transparent superhydrophobic gel nanocoating with self-healing properties. Chem. Commun..

[28] Guedri L., Ben-Amor S., Gardette J., Jacquet M., Rivaton A. (2005). Lifetime improvement of poly(ethylene naphthalate) by ZnO adhesive coatings. Polym. Degrad. Stab..

[29] Guedri-Knani L., Gardette J., Jacquet M., Rivaton A. (2004). Photoprotection of poly (ethylene-naphthalate) by zinc oxide coating. Surf. Coat. Technol..

[30] Peternel I.T., Koprivanac N., Božić A.M.L., Kušić H.M. (2007). Comparative study of UV/TiO2, UV/ZnO and photo-Fenton processes for the organic reactive dye degradation in aqueous solution. J. Hazard. Mater..

[31] Chakrabarti S., Chaudhuri B., Bhattacharjee S., Das P., Dutta B.K. (2008). Degradation mechanism and kinetic model for photocatalytic oxidation of PVC–ZnO composite film in presence of a sensitizing dye and UV radiation. J. Hazard. Mater..

[32] Parida K., Parija S. (2006). Photocatalytic degradation of phenol under solar radiation using microwave irradiated zinc oxide. Solar Energy.

[33] Tankhiwale R., Bajpai S. (2012). Preparation, characterization and antibacterial applications of ZnO-nanoparticles coated polyethylene films for food packaging. Colloids Surf. B Biointerfaces.

[34] Faraj M., Ibrahim K., Ali M. (2011). PET as a plastic substrate for the flexible optoelectronic applications. Optoelectron. Adv. Mater..

[35] Keskin G., Kızıl G., Bechelany M., Pochat-Bohatier C., Öner M. (2017). Potential of polyhydroxyalkanoate (PHA) polymers family as substitutes of petroleum based polymers for packaging applications and solutions brought by their composites to form barrier materials. Pure Appl. Chem..

[36] Yang Q., Wang J., Zhang S., Tang X., Shang G., Peng Q., Wang R., Cai X. (2014). The properties of poly(3-hydroxybutyrate-co-3-hydroxyhexanoate) and its applications in tissue engineering. Curr. Stem Cell Res. Ther..

[37] Vandewijngaarden J., Murariu M., Dubois P., Carleer R., Yperman J., Adriaensens P., Schreurs S., Lepot N., Peeters R., Buntinx M. (2014). Gas Permeability Properties of Poly(3-hydroxybutyrate-co-3-hydroxyhexanoate). J. Polym. Environ..

[38] Ragaert P., Buntinx M., Maes C., Vanheusden C., Peeters R., Wang S., D’Hooge D.R., Cardon L. (2019). Polyhydroxyalkanoates for Food Packaging Applications. Reference Module in Food Science.

[39] Ultrasonic Spray Coating of Nanoparticles. http://www.sono-tek.com/wp-content/uploads/2018/07/Ultrasonic-Spray-of-Nanoparticles.pdf.

[40] Pham N.P., Burghartz J.N., Sarro P.M. (2005). Spray coating of photoresist for pattern transfer on high topography surfaces. J. Micromech. Microeng..

[41] Pham N.P., Boellaard E., Burghartz J.N., Sarro P.M. (2004). Photoresist coating methods for the integration of novel 3-D RF micro-structures. J. Microelectromech. Systems.

[42] Lang R.J. (1962). Ultrasonic Atomization of Liquids. J. Acoust. Soc. Am..

[43] Alternative Energy & Nanomaterials. https://www.sono-tek.com/industry/alternative-energy-nanomaterials/cnt-nanowires-and-other-nanomaterials/.

[44] Slegers S., Linzas M., Drijkoningen J., D’Haen J., Reddy N., Deferme W. (2017). Surface Roughness Reduction of Additive Manufactured Products by Applying a Functional Coating Using Ultrasonic Spray Coating. Coatings.

[45] How Ultrasonic Nozzles Work. https://www.sono-tek.com/ultrasonic-coating/how-ultrasonic-nozzles-work/.

[46] Bose S., Keller S.S., Alstrøm T.S., Boisen A., Almdal K. (2013). Process Optimization of Ultrasonic Spray Coating of Polymer Films. Langmuir.

[47] Scanning Electron Microscope A To Z. https://www.jeol.co.jp/en/applications/pdf/sm/sem_atoz_all.pdf.

[48] Abbas M., Buntinx M., Deferme W., Reddy N., Peeters R. Comparison of the oxygen gas and UV barrier properties of nano ZnO coated PET and PHBHHx packaging films via ultrasonic spray coating. Proceedings of the Nanotech France 2019 International Conference.

[49] Marra A., Rollo G., Cimmino S., Silvestre C. (2017). Assessment on the Effects of ZnO and Coated ZnO Particles on iPP and PLA Properties for Application in Food Packaging. Coatings.

[50] Chang H., Wang Z., Luo H., Xu M., Ren X., Zheng G., Wu B., Zhang X., Lu X., Chen F. (2014). Poly(3-hydroxybutyrate-co-3-hydroxyhexanoate)-based scaffolds for tissue engineering. Braz. J. Med. Biol. Res..

[51] Spectrophotometry. https://chem.libretexts.org/Bookshelves/Physical_and_Theoretical_Chemistry_Textbook_Maps/Supplemental_Modules_.

[52] Zak A.K., Razali R., Majid W.H.B.A., Darroudi M. (2011). Synthesis and characterization of a narrow size distribution of zinc oxide nanoparticles. Int. J. Nanomed..

[53] Sahare S., Veldurthi N., Singh R., Swarnkar A.K., Salunkhe M., Bhave T. (2015). Enhancing the efficiency of flexible dye-sensitized solar cells utilizing natural dye extracted fromAzadirachta indica. Mater. Res. Express.

[54] The Beer-Lambert Law. https://chem.libretexts.org/Bookshelves/Physical_and_Theoretical_Chemistry_Textbook_Maps/Supplemental_Modules_(Physical_and_Theoretical_Chemistry)/Spectroscopy/Electronic_Spectroscopy/Electronic_Spectroscopy_Basics/The_Beer-Lambert_Law.

